# Effectiveness and Efficiency of Persuasive Space Graphics (PSG) in Motivating UK Primary School Children’s Hand Hygiene

**DOI:** 10.3390/ijerph17072351

**Published:** 2020-03-31

**Authors:** Sophie Rutter, Catherine Stones, Jane Wood, Colin Macduff, Margarita Gomez-Escalada

**Affiliations:** 1 Information School, the University of Sheffield, Sheffield S1 4DP, UK; 2 School of Design, University of Leeds, Leeds LS2 9JT, UK; j.wood@mihealth.info; 3 School of Design, Glasgow School of Art, Glasgow G3 6RQ, UK; c.macduff@gsa.ac.uk; 4 School of Clinical and Applied Sciences, Leeds Beckett University, Leeds LS1 3HE, UK; m.gomez-escalada@leedsbeckett.ac.uk

**Keywords:** handwashing, hand hygiene, children, schools, measures, data collection tools, research methods

## Abstract

Good hand hygiene is necessary to control and prevent infections, but many children do not adequately wash their hands. While there are classroom communications targeted at children, the toilet space, the location of many hand hygiene activities, is neglected. This paper describes an initial evaluation of “123” persuasive space graphics (images and messages integrated within an architectural environment that encourage specific actions). The effectiveness (whether hand hygiene improves) and efficiency (the ease with which a setting can adopt and implement an intervention) is evaluated in three UK schools and one museum. Five evaluations (participant demographic, handwashing frequency, handwashing quality, design persuasiveness, stakeholder views) were conducted. In the school settings, persuasive space graphics increased the quality and frequency of handwashing. In the museum setting, frequency of handwashing slightly increased. In all settings children found the graphics persuasive, and stakeholders also believed them to be effective. Stakeholders considered persuasive space graphics a low-cost and time-efficient way to communicate. It can be concluded that persuasive space graphics are effective in increasing hand hygiene, particularly in school settings where children have a longer exposure to the graphics. Persuasive space graphics are also an efficient low-cost means of communicating hand hygiene.

## 1. Introduction

Infection prevention and control is a key strategy for tackling antimicrobial resistance in the UK’s five-year action plan [1] and twenty-year vision [2]. Washing hands is thought to be the most effective action a person can take to reduce risk of infection-related illness [3]. It is estimated though that in the UK only 52% of people (19% in the world) wash their hands with soap after using toilet facilities [4]. It is, therefore, vital that the importance of hand hygiene is effectively communicated and that this communication results in long lasting change [4].

Children are a key target of hand hygiene promotions. They are particularly prone to infections because their immune systems are still developing. Sickness in children raises the risk of infection within households. It also increases absenteeism. This adds pressure to school systems and impacts children’s learning. Additionally, parents need to take leave to care for sick children, and there is an increase in general practitioner visits [5]. Another reason for promoting hand hygiene to children is that habits formed in childhood tend to be retained for life [6]. Moreover, once children are educated in handwashing, they in turn promote hand hygiene to the wider community [1].

**Figure 1:**
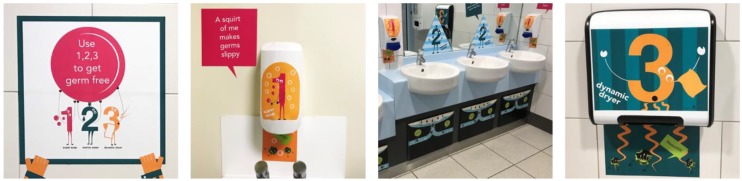
Example “123” persuasive space graphics (PSG).

In this study, the effectiveness and efficiency of persuasive space graphics (PSG) in motivating handwashing in English primary school toilets are evaluated. PSG are graphics integrated within an architectural environment to encourage specific actions. The PSG in this study are based around the concept of “use 123 to get germ free”, where 1 is the soap, 2 is water and 3 is the drying device. Hand hygiene messages and graphics are integrated within the soap dispenser, taps and hand dryer (see Figure 1). Graphics are also placed in cubicle areas and surrounding wall space. A co-design methodology was used to develop the PSG; the theoretical basis for this will be reported in future work. The designs will become available for schools to download and print from [7].

Globally many interventions have been targeted at children; those most notable in the UK include Hands up for Max!, e-Bug, and on a smaller scale, Glo-yo. Hands up for Max! was developed by the former Health Protection Agency (now Public Health England) and provides educational resources (lesson plans, a six-minute animation, posters on how to wash hands and stickers) to primary schools. In a cluster randomised controlled trial (178 schools) using school supplied absence data, Hands up for Max! was primarily evaluated for whether it reduced absences. In addition, employing children focus groups, teacher interviews and observations, eight of these schools were also selected to participate in a sub-study of hand hygiene knowledge, attitude and behaviours, and an assessment of how the schools implemented the intervention. It was found that Hands Up for Max! increased knowledge and awareness, but this did not lead to a reduction in absences [8]. The study’s authors also found that educating children on hand hygiene, while necessary, is not in itself sufficient. Structural factors such as the time available to wash hands and the cleanliness of facilities are also key [8]. A related evaluation found low take up of the intervention beyond the trial; for successful implementation, educational resources need to be embedded into the curriculum [9].

e-Bug [10], a European project led by Public Health England with a consortium of 28 partner countries, aims to educate children about hand and respiratory hygiene, as well as the prudent use of antibiotics to combat antibiotic resistance [11]. e-Bug provides primary and secondary school teachers and students with educational packages that include lesson plans and activities. Resource packs were posted to every primary (*n* = 19,142) and secondary (*n* = 5637) school in England in 2010 and again in 2015. Sent out with the 2015 packs was an invitation for educators to take part in an online survey evaluating the resources. Of the 695 respondents (2.8% response rate), 94% rated the resource as either good or excellent [12]. However, given the low response rate these results need to be interpreted with some caution. In an earlier evaluation (conducted in England, France and Czech Republic) the effectiveness of the packs was measured by assessing children’s (aged 9–11 and 12–15) knowledge gain when using e-Bug. There was little difference in knowledge gain between e-Bug schools and those following the usual curriculum [13]. 

Glo-yo is a small yo-yo like device that incorporates an educational video and dispenses iridescent soap that can indicate how well hands have been washed under UV light (also incorporated into the device). It was developed at the University of Nottingham, with children (aged 5–8) from two primary schools coming up with the initial designs. It was evaluated at the same two schools using children’s self-reports of handwashing, microbial sampling of their hands, and reports from parents and teachers. Although no significant difference was found in microbial load on children’s hands, a 34% increase in handwashing was reported. In follow-up interviews a year later, teachers and children also reported a sustained improvement in handwashing [14].

Both Hands up for Max! and e-Bug are classroom-based interventions that need to be delivered by teachers. This puts a demand on teacher time and the interventions are asynchronous and geographically separate from the action of handwashing. Point-of-decision signs have been effective in changing behaviour in many studies [15]. In our study, like Glo-yo [14], children helped to co-design communication to be placed at the point-of-decision (i.e., in toilet facilities). Unlike Glo-yo where a new product is introduced into the toilet facilities, the PSG are incorporated into existing facilities (see Figure 1).

To develop hand hygiene promotions that are both economical and impactful, it is necessary to understand the effectiveness and efficiency of interventions. Effectiveness is the degree to which an intervention achieves a desired outcome (in this case an increase in hand hygiene), whereas efficiency is how successful an intervention is compared to other interventions [16]. Efficiency goes beyond comparing the effectiveness of interventions (as this is usually measured under ideal conditions) to how well an intervention can be adopted and implemented in real-life settings [16]. The research questions for this evaluation are

1. How effective are “123” persuasive space graphics (PSG) at motivating hand hygiene?
2. How efficient are “123” persuasive space graphics (PSG) in communicating hand hygiene?

Before conducting a major roll-out of any intervention it is prudent to do a smaller-scale test [17]. In this study, the PSG are evaluated in four settings. Three of the settings are the UK primary schools (children aged 4 to 11) where the designs for the PSG were created and the fourth setting is a UK national children’s museum, which is a project partner. 

## 2. Evaluation Frameworks

Frameworks offer a systematic way to analyse interventions and help to incorporate all the aspects that evaluations should address. They can also help ensure that key implementation strategies are incorporated into future dissemination plans [18]. There are a variety of frameworks and tools that can be used, such as RE-AIM (reach, effectiveness, adoption, implementation and maintenance) [19], PRECIS-2 (PRagmatic Explanatory Continuum Indicator Summary) [20], PRISM (Performance of Routine Information System ManagementO [21], CFIR (Consolidated Framework for Implementation Research) [22], TREND (Transparent Reporting of Evaluations with Non-randomized Designs) [23], and MRC (Medical Research Council) evaluation framework [24]. All these frameworks are functional, comprehensive and could be used to evaluate this study. RE-AIM was selected as the primary framework because it could be easily applied to the school setting. Moreover, it could be used to evaluate this small-scale intervention, while at the same time informing further development of the intervention for future dissemination to more settings. 

RE-AIM offers a comprehensive evaluation framework that can be used to evaluate and design public health interventions across five dimensions (reach, effectiveness, adoption, implementation and maintenance). The dimensions operate at setting level (adoption, implementation and maintenance) and/or participant level (reach, effectiveness and maintenance).

- “Reach” is a measure of the representativeness of individuals who have agreed to participate in an intervention.
- “Effectiveness” (referred to as “Efficiency” in some versions of the framework) is a measure of targeted outcomes including quality of life, economic costs and unintended negative consequences.
- “Adoption” is a measure of the proportion and representativeness of settings that agree to the intervention.
- “Implementation” is a measure of the degree to which settings deliver interventions as intended.
- “Maintenance” is a measure of whether individuals sustain behaviour change and whether organisations continue to deliver the intervention [25,26].

Taken together, the five dimensions offer a holistic evaluation of the impact of an intervention [25]. RE-AIM is usually used to evaluate interventions that have taken place, but it can also be used to aid the development and design of interventions [26]. As this stage in this study, the evaluation is an initial test of the designs in the three co-design schools and partner museum. The framework is firstly used to evaluate the intervention as it is, primarily its effectiveness (RQ1). Then efficiency is examined by considering the potential for wider reach, adoption and implementation (RQ2) in a larger roll-out to more schools and more settings. Maintenance and the potential for long-term impact are also considered (RQ1 and RQ2).

## 3. Overview of Evaluation Design

### 3.1. Evaluation Methods

**Figure 2:**
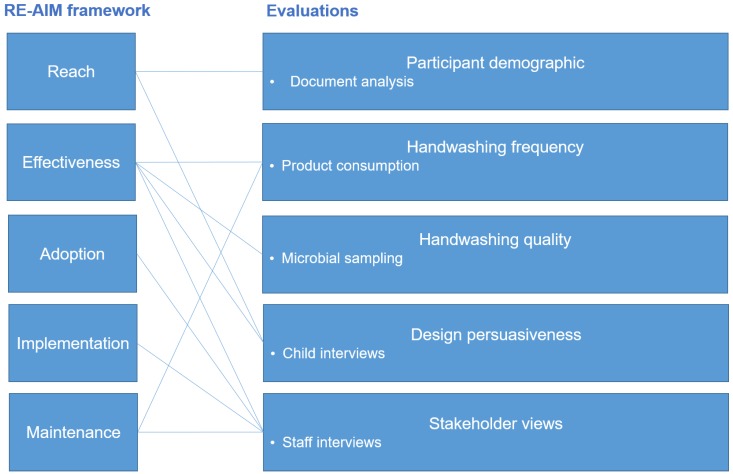
Overview of evaluation methods.

Both quantitative and qualitative research methods are necessary to evaluate health interventions [16] because “public health interventions are complex and do not conform to a simple input-output model” [27]. In this study, five evaluations with diverse methods are employed: participant demographic (document analysis), handwashing frequency (counting product consumption), handwashing quality (counting microbial presence), design persuasiveness (child interview study) and stakeholder views (staff interview study). How the evaluations map onto the RE-AIM framework is illustrated in Figure 2.

### 3.2. Participants

To better understand the influence of the setting on the effectiveness of PSG, different settings were selected so that comparisons could be made. Three co-design schools and a partner museum participated in this evaluation (see Table 1). The settings and the recruitment of these settings are described in detail in Section 4. Each evaluation describes which settings were selected to take part in particular evaluations and why. In the school settings, handwashing frequency (evaluation 2) was a whole school evaluation, handwashing quality (evaluation 3) was evaluated with one class from each of the year groups from year 1 to year 6 (ages 5–11), and design persuasion (evaluation 5) was evaluated with any and all children available to participate at the time of data collection. The evaluations were conducted at different times in the museum setting so it is likely that different children participated in each evaluation.

The PSG were installed by the research team in one set of boys’ and girls’ toilets in each setting in spring 2019. The PSG are still in situ at the time of writing this publication.

### 3.3. Ethics

All subjects gave their informed consent for inclusion before they participated in the study. The study was conducted in accordance with the Declaration of Helsinki, and the protocol was approved by the Ethics Committee of PVAR 17-004. on August 2017 and November 2018. To ensure confidentiality neither the names of the settings, nor those of adult and child participants were recorded. How participants were recruited and consent received are described for each evaluation under the respective sections.

## 4. Evaluation 1: Participant Demographic

The demographic characteristics of the participating settings are identified to evaluate the characteristics of children the PSG have reached in this study.

### 4.1. Methods

In this evaluation an analysis of publicly available documents was conducted to identify the demographic characteristics of participant settings.

#### 4.1.1. School and Museum Recruitment

Schools were recruited for both the co-design and evaluation phases of the project. All 27 local authority schools were sent an email asking if they wanted to take part in a “creative project to improve hand hygiene in school toilets”. Three schools responded and, following further contact, two head teachers agreed to their setting participating. Although the response rate was low, this could be expected given the time commitment required of the schools. To ensure different catchment area demographics (see Table 2), the head teacher of a third school, outside the area, known to C.S. was also contacted and became the third school partner. The three schools selected to participate in the co-design continued to participate in the evaluation. To further ensure that the PSG reached a diverse audience, a national children’s museum known to C.S. was approached to take part in the installation of PSG and evaluation.

#### 4.1.2. Data Collection

Publicly available reports were used to identify the demographic characteristics of children attending the participating schools and partner museum. For the partner museum annual reports and other documents were downloaded from their website. For the three schools Ofsted inspection reports were used [28]. Ofsted inspects English schools at regular intervals and makes an overall judgement of whether they are outstanding, good, require improvement or inadequate. Included in full inspections is a description of the school, demographic data on children attending the school and the proportion of children who are eligible for free school meals. Free school meals are considered an indicator of socio-economic disadvantage [29].

### 4.2. Results

The demographic characteristics of the settings are summarised in Table 2. 

### 4.3. Limitations

This evaluation relied on publicly available documents that describe the demographic characteristics of the participating settings. These can be used to see how representative the study settings are in comparison to the wider population. However, this analysis only indicates who the PSG potentially reached, and not who they did reach within the settings. 

### 4.4. Evaluation Summary and Conclusion

All four settings cater to children aged 4–11, with the partner museum also catering to younger children. Together the schools and the museum are attended by children from a range of ethnic backgrounds and socio-economic statuses. All the settings are located in the north of England and Midlands and are thus geographically homogenous. Only schools judged as good by Ofsted participated in this study.

It can be concluded that:

- the evaluation in this study is potentially transferable for children aged 4–11 from the north of England irrespective of socio-economic status and ethnic background;
- further piloting is required to determine transferability of the PSG to other regions and poor performing schools.

## 5. Evaluation 2: Handwashing Frequency

The frequency of handwashing is measured to evaluate the effectiveness and maintenance of the PSG. 

### 5.1. Methods

Product consumption has been employed as a proxy measure of hand hygiene both in the community and schools [30]. In these studies, a higher consumption of a handwashing product is considered indicative of greater hand hygiene. For example, soap consumption increased by up to 10% after hand hygiene messages were installed in motorway service stations [17].

#### 5.1.1. Participants and Data Collection

All four settings participated in the monitoring of soap consumption and School 3 also participated in drying consumption. Data were collected for both pre- and post-PSG installation periods. For practical reasons (such as access), the participating settings collected the consumption data and so the duration of data collection depended on the good will of the staff at the settings. The research team requested a minimum of four weeks for each data collection period. School 1 and 3 collected data for two consecutive four-week periods post-installation. In School 3, the designs were damaged during the second four weeks and were reinstalled. Data were then collected for a third four-week period. School 2 misplaced some of the datum. Since this risks invalidating the results, consumption data are not reported for School 2. 

The process of soap data collection depended on the method used to keep soap dispensers operational in the different settings. The participating schools topped up soap dispensers from a large container with the same container used to fill all soap dispensers within the school. To minimally disrupt staff procedures, schools were asked to keep a record of how much soap was consumed overall. This meant that the difference in soap consumption between pre- and post-PSG installation could be measured; yet where schools had more than one set of toilets it was not possible to differentiate between toilets where PSG were/were not installed. 

The partner museum replaced packets of soap in each dispenser when they were nearly empty. The museum was asked to store and label the empty packets. In this way it was possible to differentiate soap consumption for toilets where PSG were/were not installed. This was crucial as children may only visit one toilet. Moreover, measuring consumption in both sets of toilets meant that fluctuations in visitor numbers (that are less likely in a school setting) could be accounted for. Each toilet in the museum has two or three dispensers, with one of these dispensers set at a lower level for children. To record differences in children’s (rather than adults’) soap consumption, data are reported for the lower dispensers only. It is recognised that children may also use the higher dispensers and indeed adults may use the lower one. The counting of soap packet changes commenced after the first full replacement (i.e., a change was only counted if a full packet was consumed). When full, the packets weighed 1.1 kg. Some soap remained in the empty packaging (mean 83 g, SD 9.4 g). To simplify calculations, the difference in weight between empty packets is not incorporated into the evaluation.

Drying consumption was only measured in School 3 because the drying system in this school (paper towels) was the only system that could be measured. Roll towels (School 1) were often found run through by the middle of the day, and an affordable method to measure electric dryer consumption (partner museum and School 2) could be not be identified. 

#### 5.1.2. Data Analysis

To evaluate consumption, a percentage change in consumption between pre- and post-installation is reported. Statistical significance was not tested since the data collection method used means that there are not enough data points to reliably conduct such tests.

### 5.2. Results

The results show a large increase, between 41% and 60%, in soap consumption in the schools following design installation (Table 3). During the second post-installation period at School 3, children had removed designs from the toilets and soap consumption remained close to pre-PSG levels, suggesting that the increase in handwashing frequency was not maintained when the PSG were absent. Whether this is because the initial installation period was relatively short, or the PSG are a necessary “call to action” should be investigated in future work. Drying consumption at School 3 initially decreased by 5% in post-installation period I, and then increased by 15% in post-installation period II and by 18% in post-installation period III. Although this shows that towel consumption increased overall, the inconsistency of the result across the different time periods means it is not possible to confidently link this to the PSG. 

In the partner museum, footfall was generally higher in the PSG-free toilets, which were located next to the cafeteria, since overall soap consumption was greater in these toilets (Table 4). Soap consumption increased in the post-installation period in both sets of toilets (PSG, PSG-free). This is attributable to an unconnected increase in visitor numbers. However, consumption increased the most in the toilets featuring PSG, indicating that proportionately more children washed their hands when the PSG were present. It should be noted though that the increase in frequency of handwashing that can be attributed to the presence of the PSG is less than in the school settings.

### 5.3. Limitations

The amount of data that could be collected was limited as the research team was reliant on the goodwill of the staff at the settings to provide the data. It was also necessary to adapt data collection methods for the varying facilities in the different settings, making comparisons across settings difficult. More automated methods could lessen the burden on settings, standardise the data collected and provide more data points that would allow for statistical analysis. As technology develops it is likely that this could be employed in future studies.

The data collection periods, while adequate to identify whether the introduction of PSG increased handwashing, should be extended to identify whether the increase in handwashing is maintained long-term. 

### 5.4. Evaluation Summary and Conclusion

It can be concluded that:

- PSG have been very effective in increasing handwashing frequency in the school settings. Soap consumption increased between 41% and 60% when the PSG were present.
- handwashing frequency was not maintained when the PSG were absent. Whether longer term exposure to the PSG would help maintain behaviour change when the designs were present and/or absent needs to be tested in future work.
- the PSG were moderately effective in increasing handwashing frequency in the museum.

## 6. Evaluation 3: Handwashing Quality

Handwashing quality is measured to evaluate the effectiveness of the “123” PSG. 

### 6.1. Methods

The presence of microorganisms on the body (usually hands) has been used as a proxy measure in studies of school children to indicate handwashing quality [30].

In School 1 a pre-test was conducted on 14 February 2019. PGS were installed on 2 April 2019 and the post-test was conducted on 24 April 2019. To be certain that it was not simply a change in the toilet environment that led to a change in hand hygiene a gap of 22 days was left between PSG installation and the post-test. In the museum the pre-test was conducted on 4 January 2019. The designs were installed 8 March 2019 and the post-test was conducted on 9 March 2019. For the museum it was not necessary to leave a gap between the installation of PSG and the post-test as most visitors would not be aware that the environment had changed. 

#### 6.1.1. Participants and Recruitment

The research team asked School 1 to recruit a spread of year groups (Table 5). The school selected classes (from year 1 through to year 6 with children aged between 5 and 11) and sent letters home to parents/carers informing them of the study with the option to opt their children out (none elected to do so). Children from each participating class were then asked if they would like to take part (all wished to do so). At the partner museum children aged between 4 and 11 were recruited (Table 5). Other children (notably young siblings of older children) that wanted to participate could take part, but their results were not analysed. To obtain consent from parents, participants were recruited during the school holidays (pre-installation) and on a weekend (post-installation). 

#### 6.1.2. Procedure and Data Collection

**Figure 3:**
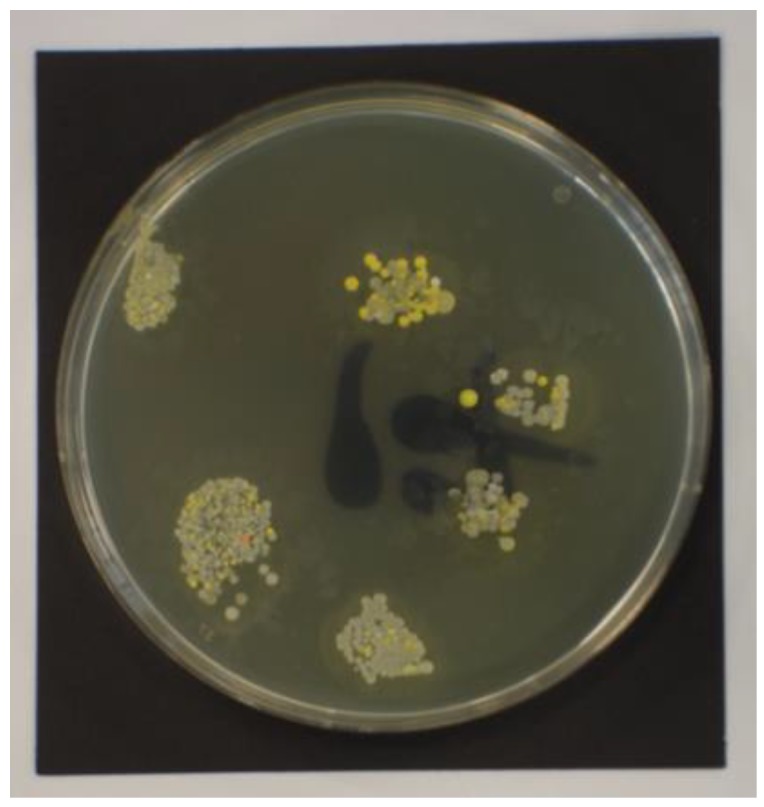
Example of a child’s fingerprint on an agar plate.

To sample the microorganisms on children’s hands, prints of the finger pads and thumb were taken on agar plates (Figure 3). When the samples were taken was dependent on the idiosyncrasies of the individual settings. 

In schools, as most toilet use is during break times, sampling multiple children as they left the toilets is impracticable. Additionally, news of the sampling would likely spread, and children could be prompted to wash their hands. For those reasons, at School 1 all children were sampled at the same time after their lunch break. Members of the research team went into each classroom and children who wished to take part lined up to have their prints taken. This approach allowed for analysis of the impact of the PSG on the cleanliness of children’s hands at a specific time point. Children were asked when they had last used the toilet, but many could not remember so data cannot be tied to a specific toilet event.

At the museum, the research team set up a data collection table at a discrete distance from the toilets. One member of the research team stayed at the table and collected data, while another member recruited children from outside the toilets. When a child exited the toilet their parent/carer was informed about the study and permission was asked to approach the child. If the child was interested in participating, they were escorted to the data collection table where their parent/carer were asked to sign a consent form. This approach meant that it was possible to analyse how effectively children had washed their hands after using the toilets. However, as the data collection took place in a public space some of the children who participated were possibly aware of the study prior to using the toilets, and this also could have influenced how well they washed their hands. However, the circumstance was the same for both pre- and post-installation samples and therefore the results are comparable. 

#### 6.1.3. Plate Preparation and Incubation

Modified Letheen Agar plates (manufactured by Becton Dickinson, Fraklin Lakes, NJ, USA) were prepared following manufacturer’s instructions. Following sampling the plates were incubated for 24 h at 37 °C and then refrigerated at 4 °C until the plates could be photographed (a maximum of one week).

#### 6.1.4. Data Analysis

The plates were analysed from the photographs. For five plates from the school post-installation sample colonies were undistinguishable. The remaining colonies were counted using Image J [31], freely available through the NIH (US National Institute for Health). The colonies were counted using the multi-point tool as a counter. Altogether 81,922 colonies were found and counted for the 392 plates collected.

First the spread of colony counts was checked across the samples before and after for both settings using box and whisker plots in SPSS v22 (IBM Corp., Armonk, NY, USA). The data are not normally distributed (Shapiro–Wilk normality test). Regardless, t-tests are considered more appropriate than equivalent non-parametric tests for large samples even when the data are not normally distributed [32,33]. To find out if the PSG encouraged children who were previously not washing their hands, a one-tailed t-test was conducted to identify if there was a significant reduction in high colony counts between pre- and post-tests [34].

### 6.2. Results

**Figure 4:**
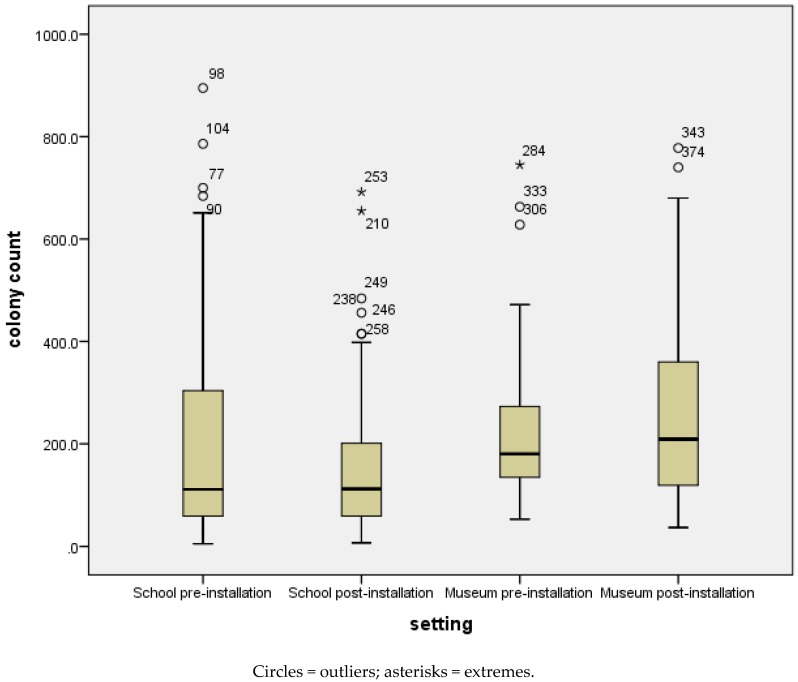
School 1 and museum agar plate results.

Results are shown in Figure 4. For both settings, the data are skewed towards a high colony count suggesting that many children in the sample did not have good hand hygiene practices. In all conditions the outliers are for high colony counts suggesting that some children had very poor hand hygiene practices. For school 1, the number of samples with high colony counts decreased after installation of the PSG (from a mean colony count of 186 to 151 and a median colony count of 111 to 112). An independent sample one-tailed t-test indicated that this is statistically significant t(270), 1.861, *p* = 0.032. This could suggest that children who previously either did not wash or did not wash their hands well improved their hand hygiene practices. However, in the museum setting the spread increased post-installation (from a mean colony count of 220 to 260 and a median colony count of 181 to 209). The result is not statistically significant. This could be explained by the fact that, unlike the school settings, different children participated in the pre- and post-installation sample. In addition, children in the museum only had single exposure to the PSG. 

### 6.3. Limitations

The idiosyncrasies of the settings meant that different methods were necessary for School 1 and the museum. It is therefore not possible to make direct comparisons between settings. 

To reassure children that they could not be identified from the agar plates no personal data were collected. Therefore, it is not possible to align pre- and post-installation samples, and it is only possible to ascertain whether hand hygiene generally increased in settings. This is particularly problematic in the museum setting where different children participated in the pre- and post-installation sample. As many of the museum participants visit only once, it would not be practical or feasible to collect data from the same children.

Another consideration is the timing of the test. In the schools it was necessary to test all children at the same time otherwise news of this test could prompt a change in behaviour. As microorganisms are present in the environment, if there is a gap between a handwashing opportunity and the test, it may be unclear if the hands were washed or that a child simply picked up new microorganisms. Even if there is no gap between a handwashing opportunity and the test, results may be affected by the preceding event (e.g., defecation vs. urination) [30]. Even though there are limitations, testing microbial load is still a key evaluation, as it is the only measure that irrefutably indicates that effective handwashing has taken place.

### 6.4. Evaluation Summary and Conclusion

It can be concluded that:

- the “123” PSG were effective in increasing handwashing quality in the school settings;
- the “123” PSG were not effective in increasing handwashing quality in the museum setting.

## 7. Evaluation 4: Design Persuasiveness

How persuasive the “123” PSG are in encouraging hand hygiene was evaluated by investigating whether the PSG reached children and how effective the children considered the PSG to be.

### 7.1. Method

As handwashing is a social norm [17] simply asking children if they have washed their hands is unlikely to lead to reliable and credible answers. Moreover, there are different stages to persuasion. To evaluate whether the designs have been effective McGuire’s [35] communication–persuasion matrix, a general framework for considering behaviour change, was employed. McGuire [35] describes that for a message to persuade, a person must (1) be exposed to a message; (2) attend to this message; (3) engage with the message; (4) understand the message; (5) relate the message to what is already known; (6) acquire the skills to comply with the message; (7) accept the message; (8) the message must be retained in the memory; (9) the message must be retrieved from memory; (10) a decision must be made on whether to comply with the message; (11) the decision must be acted on; and finally, (12) a new pattern of behaviour must be established.

As participants were children (aged 4–11) asking them a question for each of McGuire’s (1985) twelve steps could be confusing, so four steps that were most relevant to this study were selected. As the PSG were situated in the toilet environment at the point-of-decision, McGuire’s (1985) steps of (1) exposure, (8) retain message and (9) retrieve message were considered less critical. Moreover, in a review of studies that used pictures to communicate health information, Houts et al. [36] found that, to varying degrees, pictures can increase attention (step 2), comprehension (step 4), recall (step 9), and adherence (step 11). These steps were therefore selected. Agree with the message (step 7) was also selected.

#### 7.1.1. Participant Recruitment and Data Collection Procedure

In total, 80 girls and 54 boys were interviewed from the three participating schools and partner museum (Table 6). A balanced sample was strived for, but this was inevitably constrained by the settings, and the convenience and keenness of our participants. 

Each child was interviewed individually by J.W. The interviews were audio recorded and transcribed verbatim. At the partner museum children were recruited after leaving the toilets. Parents were informed about the study as they exited the toilets with their child. If parents were happy then J.W. approached each child and explained what participation would entail including that they could withdraw at any time.

In the schools, as all children were familiar with the designs, they were interviewed during their lunch break. Participating children helped to recruit other children with minimal disruption to school activities. Prior to data collection, the participating schools sent letters home to parents/carers informing them of the study, the activities involved and the option to remove their child from participating. No parent/carer chose to withdraw their child from the study. On the day of data collection, J.W. explained in person to each child what participation would entail including that they could withdraw at any time. All children assented to participating in the study. 

#### 7.1.2. Interview Design and Analysis

The interview questions are based on the four steps selected from McGuire’s (1995) twelve-step persuasion communication matrix (Table 7). For clarity, the steps in this paper were renumbered and mapped onto the RE-AIM framework.

For the steps to make sense to our young audience they were rephrased into questions with simpler language. This was particularly the case for step 3 (acceptance of arguments) as this phrase was too abstract for young children to understand. Acceptance of arguments was rephrased as “trust”, but it is recognised that the phrases are not exact equivalents. In order for responses to be quantified the questions were closed for steps 1, 3 and 4. For step 2 (comprehension) simply asking children if they understood the designs could be very misleading as what children understood may not be what was intended. Instead they were asked to say what they found out (Q2.1), and then their answers were checked for whether they had correctly understood this. Whether children understood the key message (“123”) was also checked (Q2.2). To further illuminate how and why the designs persuaded, additional open questions were asked for steps 3 and 4. 

A combination of quantitative and qualitative techniques was used to analyse the interview data. For the closed questions (Q1, Q3.1 and Q4.1) the number of yes/no/do not know responses was counted. To quantitatively analyse the persuasiveness of designs for step 2 (comprehension), children’s understanding of the 123 phrase was rated by J.W. and verified by C.S. (Q2.1 and Q2.2). Using the Chi-squared statistical test (SPPS v26), each step was checked for differences between settings, year groups and sex. Whether comprehension (step 2) had a bearing on attitude change (step 4) was tested. 

A team approach was taken to analyse the qualitative data for Q3.2 and Q4. An initial categorisation was carried out by J.W. C.S. further refined the categories, and then S.R. reviewed all categorisations. Discussion continued between S.R., C.S. and J.W. until 100% agreement was reached. For Q3.2 the data was initially analysed inductively and then mapped deductively onto pre-defined trust constructs already identified from prior research [37]. For Q4 the categories were developed inductively as no a priori categorisation existed, since how hand hygiene attitudes could change is particular to this study.

### 7.2. Results

**Figure 5:**
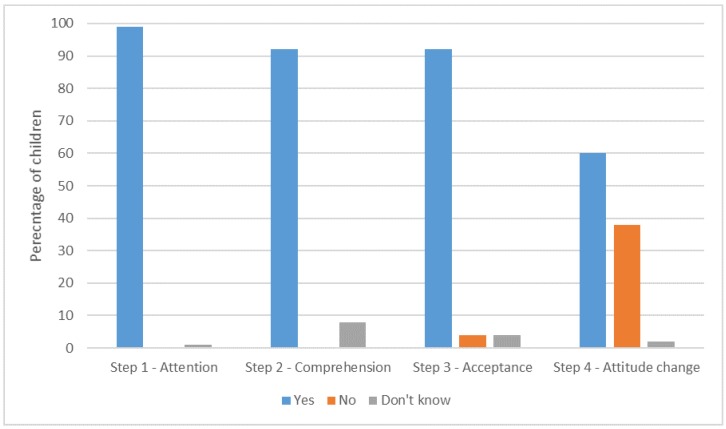
Design persuasiveness results.

The design persuasiveness results are shown in Figure 5. 

#### 7.2.1. Step 1: Attention (Reach and Effectiveness)

In total, 99% (132/134) of children attended to the designs. 

#### 7.2.2. Step 2: Comprehension (Effectiveness)

In total, 92% (123/134) of children understood at least one of the hand hygiene messages communicated by the PSG (Q2.1). In addition, 40% (53/134) of children understood at least one message from the designs, and 51% (69/134) understood two or more. Children reported that they found out they should wash their hands (71%), information about faeces and germs including that germs/poo make you sick (64%) and how to keep toilets clean by closing the toilet lid, and so on (14%). No miscomprehensions were reported for this question but 9% (12/134) of children could not remember or did not know what was being communicated.

Children’s comprehension was further tested by asking specifically what “123” means (Q2.2). This is a key concept of the designs that reminds children that they need to use soap, water and the dryer. In total, 47% (63/134) of children fully understood the meaning of “123” (e.g., identified all 3 elements), 18% (24/134) partly understood (e.g., identified one or two of the three elements) and 35% (47/134) did not know what “123” meant. 

The 36 children who comprehended a communication from the PSG did not understand the meaning of “123”. This suggests that some children gained an understanding of handwashing from the PSG but did not fully comprehend the entire message. The Chi-squared test was used to determine whether age, gender and setting accounted for differences in “123” comprehension. No statistical evidence for differences by age was found. However, we suspect that older children were more likely to comprehend “123”, but a larger sample size is required to give the test statistical power. There was significant evidence of an association between setting and comprehension, (χ2 (2) = 26.309, *p* < 0.001). Approximately 81% of those who fully comprehended “123” were from a school setting, whereas 63.8% of those who did not comprehend “123” were from the museum. That children in the museum had a much shorter exposure to the designs (mostly one visit), whereas children in schools were repeatedly exposed to the designs could explain this finding. There was also evidence of an association between gender and comprehension, (χ2 (2) = 6.914, *p* < 0.032) in which 66.7% of those who fully comprehended “123” were girls and only 44.7% were boys. That girls may spend longer in the toilet space than boys could account for this finding. This could usefully be investigated in further work.

#### 7.2.3. Step 3: Acceptance (Effectiveness)

“Trust” was used as a proxy for “acceptance” as more children are likely to understand this concept. About 92% (123/134) of children said that they trusted the designs (Table 8). The main reason given for trusting the designs was usefulness. This corresponds with findings in the co-design phase of this project where children told us that messages informing children about germs (rather than instruction to wash) would be effective [38]. Children also trusted “123” because it fitted with what they already knew (triangulation). The authority of the designs was also important; children thought that the institutions in which the PSG were located would not lie nor would the university that produced the PSG. Children did not give ease of use as a reason for trusting the designs, perhaps surprisingly as the PSG are at the point-of-decision. However, trust was investigated using an open question and children tended to respond with just one trust reason. Future research may helpfully establish all the reasons why children trust the PSG and the relative importance of each construct. 

#### 7.2.4. Step 4: Attitude Change (Effectiveness) 

In total, 60% (81/134) of children reported that the designs had changed their behaviour in the toilets. Positive changes included now washing hands (26), using soap and/or the dryer (35), closing the toilet lid (8), flushing the toilet (1), keeping the facilities clean (1), and no longer “messing about” (1). Two children reported a negative consequence of wanting to avoid contact with surfaces. It could be that those who did not report an attitude change did not do so because they already wash their hands, and indeed 35% (47/53) of those who stated that their attitude had not changed claimed they had washed their hands when using the toilet facilities immediately prior to the interview. 

The Chi-squared test was used to test whether age, gender, setting and comprehension of “123” accounted for differences in attitude change, but no statistically significant differences were found.

### 7.3. Limitations

Firstly, as the participants were children it was not feasible to ask them questions for each of McGuire’s steps as the differences between each step would be difficult to communicate. Some of the steps also had to be rephrased with simpler language that could be easily understood. Secondly, handwashing is a social norm and so asking participants if they have washed their hands is likely to lead to over-reporting [17]. As such it is not possible to reliably identify who washed their hands either prior to the intervention (or immediately prior to data collection). Instead children were asked more generally if their behaviour had changed (so that children were not primed). This is likely to lead to under-reporting of the persuasiveness of the PSG as it is not possible to identify whose behaviour has not changed because they already washed their hands.

### 7.4. Evaluation Summary and Conclusion

It can be concluded that:

- situating communication designs in toilets (i.e., PSG) is a powerful way to reach children, and is effective in gaining children’s attention (step 1);
- through the PSG, hand hygiene messages were comprehended (step 2) and thus successfully communicated. The core “123” message was more effective in schools where children had a longer exposure to the message. In single-visit settings, such as museums, a simpler approach may be required;
- the “123” PSG were effective in gaining children’s trust (step 3) mostly because children found the information useful and because the information triangulated with what they already knew;
- with over half of the children reporting that the designs led to a change in behaviour and nearly half either now washing their hands or using the soap and hand-dryer when they had not before, the “123” PSG can be considered effective in behaviour change (step 4), particularly as some children would already be washing their hands.

## 8. Evaluation 5: Stakeholder Views 

Stakeholders were consulted for their views on the effectiveness of the “123 PSG”, and the likely adoption, implementation and maintenance of PSG in their setting and beyond.

### 8.1. Methods

Involving stakeholders in research and evaluation has aided dissemination in several studies [39] and is important because stakeholders can help identify the applicability and costs of interventions to different settings [40]. This was particularly important for this study, as the settings that adopted the “123” PSG were the co-design schools and a partner museum, and in the current implementation the PSG were installed by the research team at no cost to the settings. A combination of focus groups and interviews was employed to gather the opinions of stakeholders from the different settings.

#### 8.1.1. Participants and Recruitment

Head teachers/primary school contacts were asked to organise a focus group with teachers who wished to provide feedback on the PSG. One focus group was held in each school. Those who were not able to attend due to other commitments (head teachers and cleaners) were interviewed separately. Cleaning staff were also approached by either the head teacher or J.W. A total of 23 stakeholders were recruited (see Table 1). The head teacher (*n* = 3) and at least one cleaner (*n* = 4) from each school were recruited as were year group teachers from School 1 (*n* = 5), School 2 (*n* = 4) and School 3 (*n* = 6). The cleaner at School 2 also works as a teaching assistant/cleaner. At the partner museum one staff member was recruited who had the most knowledge of the designs. All participants gave their voluntary informed consent.

#### 8.1.2. Interview Design and Data Collection

At the start of each session, participants were given a worksheet (so they could answer independently of each other). It featured two closed questions so that responses could be analysed quantitatively. For the remainder of the time the questions were semi-structured and answered either individually (in the case of interviews) or as a group (in the case of focus groups). A list of questions mapped onto RE-AIM was prepared in advance (Table 9) but participants were free to direct the discussion according to what was important for them.

The focus groups and interviews were audio recorded and transcribed verbatim. The quotes reported in the results were corrected for minor grammatical mistakes that often occur in conversation.

#### 8.1.3. Data Analysis

The closed questions were analysed quantitatively by counting the different responses. The open questions were analysed using qualitative content analysis, an approach that affords a scientific examination of an individual’s understanding of their social world [41] (in this study the effectiveness and efficiency of “123” PSG in the different settings). As no prior coding schemes existed, categories and codes were developed inductively (Table 10). As with evaluation 4 (children interviews) a team approach was taken to analysing the qualitative data. An initial categorisation was carried out by J.W. C.S. further refined the categories, and then S.R. reviewed all categorisation. Discussion continued between S.R., C.S. and J.W. until 100% agreement was reached. 

### 8.2. Results

**Figure 6:**
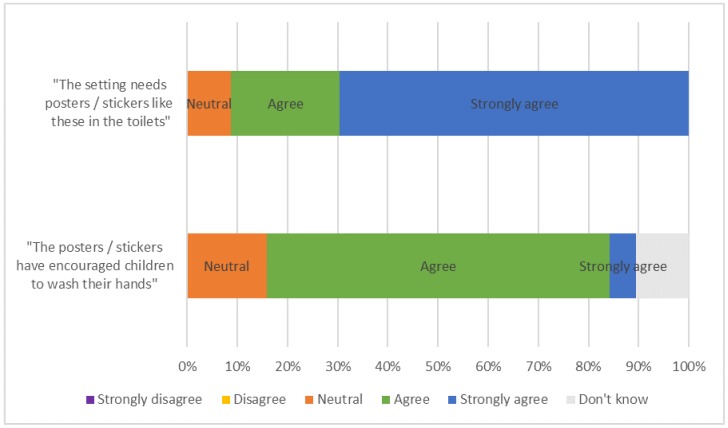
Stakeholder analysis results.

The stakeholder analysis results are shown in Figure 6.

#### 8.2.1. Engagement

Stakeholders reported that they thought children had engaged with the “123” PSG and the hand hygiene messages:

“It’s certainly created a buzz and the kids were talking about it.”

(Head teacher, School 1)

This engagement resulted in children washing their hands more, keeping toilets cleaner and asking teachers hand hygiene questions:

“Children are more alert, like they’re asking questions about them. Then, you explain to them what it is. I think it has helped them to wash their hands more.”

(Teacher, School 3)

“We’ve noticed a difference in the cleanliness of the toilets at the end of the school day. Girls, no boys in particular which surprised us. There seems to be, toilet seats seem to be down more at the end of the day and there seems to be less toilet paper on the floor, no idea why that is.”

(Head teacher, School 1)

A downside to this engagement was that when the PSG were first introduced children spent longer than was considered necessary in the school toilets. Conversely, it is anticipated that given time children could lose interest:

“Initially the children were going to the toilet more just to read the stickers and were probably spending longer in there reading the stickers than actually doing the toilet but actually since they have been there for a while its, they’re just going in there and doing what they need to do.”

(Teacher, School 1)

“I think they would work for a while and then eventually they would just become wallpaper.”

(Teacher, School 3)

#### 8.2.2. Appeal

The PSG made the toilets a visually more appealing place and even school visitors were shown to these toilets:

“It’s the fact that they are a bit bright and colourful and it makes the environment look nice because it does, because toilets are very bland environments aren’t they? So just making it a more colourful and attractive place I think has probably had a bit of an impact that we weren’t really thinking about.”

(Head teacher, School 1)

“They look bright and colourful and inviting. We’ve even had Jeremy Corbyn [leader of a UK political party] use one of them, so there you go.”

(Head teacher, School 3)

It is thought that PSG designs are interesting and fun, the messages are easy to understand for non-readers, and are professionally produced:

“Because they are so visual and they’re there all the time that they do have a really good impact and I think the fact that they are professionally done as well, I think it ups, it ups the status of it.”

(Head teacher, School 1)

However, the PSG were not thought of as appealing for adults and this could be a concern for settings where adults share facilities with children:

“I think possibly a disadvantage is it’s possibly a little too in your face maybe but that might be what the kids need whereas I’m going in as an adult.”

(Museum)

#### 8.2.3. Positioning

The location of the PSG is considered important; a distinctive feature of these hand hygiene messages is that they are situated at the point of use whereas most handwashing instruction takes place in the classroom or school hall:

“They are in the right place. That it’s all very well to teach children about hand hygiene in an assembly or in the classroom but actually this is reminding them at the very point at which it is something that they need to do.”

(Head teacher, School 3)

However, teachers would like a clearer link with the curriculum, and to also use the PSG in their classroom teaching:

“There should be a clear link to learning; personal social health education.”

(Head teacher, School 2)

#### 8.2.4. Durability

Problems with the adhesive used to stick the PSG to the walls meant that the PSG fell off the walls in all settings:

“Some were coming off. You just stick your hand up and put it back up. And on the floor when you are mopping, they were coming up, but you just stick back down afterwards.”

(Cleaner, School 2)

This also meant that it was easy for children to damage the PSG either absentmindedly or deliberately:

“It’s got to be something that doesn’t come off and that can’t be picked off because you’re always going to get inquisitive little fingers that, ooh look, there’s a sticker lets peel it off cos that’s what kids, that’s what kids do.”

(Head teacher, School 1)

#### 8.2.5. Flexibility

Stakeholders also wanted to exchange and adapt PSG. In the school settings there was concern that children could lose interest unless they were rotated:

“If we had different sets so that you know after, you know if we had three different sets one for each term then you know the message is, because otherwise it does become a bit wallpaper. So if you had three different focuses you know for autumn, spring and summer, that would, you know, just keep it fresh.”

(Teacher, School 2)

Particularly in the museum setting it was thought important that the PSG could be adapted to fit with the organisational branding:

“Possibly looking at the design just to fit in a little bit better with the museum itself, not strictly to but moving towards the way we, you know we have our branding guidelines, it just means it fits with the rest of the things we have in the museum.”

(Museum)

#### 8.2.6. Economic Cost

The PSG were funded by the project and installed by the research team, and so there was no economic cost for the participating settings. School stakeholders thought that if they had to fund themselves financial cost would be a barrier:

“We know for a fact that at the moment our budget is in absolutely dire straits there is no way that we would be able to squeeze anything out it would go by the by, couldn’t be done.”

(Teacher, School 2)

There was also some concern that schools would not be able to install PSG with the equivalent professional finish:

“Putting them in the right place as well. Little things like the toilets, the ones on the toilets, it needs to be in the right place on the toilet seat and if you get it you get it in the wrong place it’s going to, yeah so I would say putting them in the right place would be a difficult thing and getting them flat so that that don’t bubble with big stickers.”

(Teacher, School 3)

Ultimately whether settings would continue to use the designs would depend on their effectiveness. For some that they would be effective was self-evident, for others proof was needed:

“I wouldn’t need any proof. I think they’re a great idea. And if they hadn’t been funded and they were just something we could buy I would certainly consider buying them. I wouldn’t need any proof that they worked, I think they are self evidently a really great idea.”

(Head teacher, School 3)

“I’d be interested to see the results from the other schools with the use of soap, see if that’s increased. Like I have said, we have noticed a difference here but I think we would get more proof of it being effective.”

(Head teacher, School 1)

### 8.3. Limitations

Given that the interviewer had been in the schools several times and the schools had invested staff and pupil time to support the project, there may have been a tendency for interviewees to report overly positively. To mitigate this the interviewer probed for critical comments throughout the interviews. Whilst the method of interviewing led to useful insights in terms of how to develop the designs further (e.g., the need for seasonal/changeable components), certain questions relating to hand washing frequency would have been difficult to answer unless teachers were specifically monitoring the area. 

### 8.4. Evaluation Summary and Conclusion

It can be concluded that:

- PSG are effective in encouraging hand hygiene with most stakeholders strongly agreeing and none disagreeing that the PSG are required in their setting;
- PSG are effective in engaging children with the topic of handwashing and also encouraging more generally hygienic toilet practices;
- study settings adopted the designs because they are visually appealing, engaging to children and remind children what to do at the point of use;
- to increase adoption PSG should be integrated into the school curriculum and/or incorporated into classroom activities. Some flexibility in PSG selection including the ability to exchange and adapt designs would further aid adoption;
- the durability of the design material (the adhesiveness of the glue and the ease with which children could remove designs) would need to be improved for schools to adopt and maintain designs;
- evidence of the PSG working in more school settings and evidence of their long-term effectiveness would be required for schools to maintain the PSG;
- financial constraints could inhibit settings from implementation.

## 9. Discussion

### 9.1. RQ1: How Effective Are “123” Persuasive Space Graphics (PSG) at Motivating Hand Hygiene?

The evaluations indicate that the “123” PSG are persuasive (evaluation 4) and effectively increased the quality (evaluation 3) and frequency (evaluation 2) of handwashing. Stakeholders (evaluation 5) also believed the “123” PSG to be effective. The PSG are likely most effective in the school setting for which they were designed and where children had longer exposure. 

In a review of studies where pictures were used to communicate health information [36], it was found that pictures increase awareness of health messages, improve comprehension and are particularly beneficial when literacy skills are low. These aspects are likely important reasons why PSG are effective in communicating handwashing with primary school children. Primary school children (age 4–11) are developing as readers; therefore, images are a pragmatic way to communicate messages. PSG are incorporated into facilities at the point-of-decision (see Figure 1), thereby providing a timely reminder to children. Furthermore, the novelty of integrating facilities and graphics may also help attract children’s attention (particularly when compared to other forms of communication such as posters).

The PSG are also effective at reaching all children who use the facilities within a setting. In evaluation 4 (design persuasiveness) it was found that 99% of children attended to the PSG. With PSG it is possible to target all those using the facilities because the PSG are installed within the toilet environment. Individual children do not have to be identified and targeted. This is particularly helpful with hand hygiene as it is widely recognised that because handwashing is a social norm people claim to wash their hands when they do not [17]. This makes it difficult (and ethically problematic) to identify individuals for interventions.

As the “123” PSG were only recently installed at the time of these evaluations, it cannot be ascertained whether handwashing was maintained long term. However, the results of these studies can be used as a baseline in future evaluations. There is some indication in evaluation 2 (handwashing frequency) that the PSG were only effective when present but the PSG had only been installed for a relatively short time (one month). A longer exposure is likely necessary for PSG to more permanently increase hand hygiene. This should be tested in future work.

### 9.2. RQ2: How Efficient Are “123” Persuasive Space Graphics (PSG) in Communicating Hand Hygiene?

The results of the evaluations, particularly evaluation 5 (stakeholder views), indicate that PSG are an efficient way to communicate hand hygiene to primary school children, offering a low-cost alternative to classroom-based hand hygiene interventions.

An intervention effective under ideal conditions may not be effective in practice if settings struggle to implement the intervention as designed. Time constraints in the school setting make it particularly important to consider the viability of the intervention [42]. Interventions are rarely implemented as designed [42]; this has been a concern in two recent large-scale handwashing interventions directed at primary school children [9,43]. Hi Five [44] was more effective in school settings that had a high degree of implementation but none of the participating schools could fully implement the intervention as planned (lessons, mandatory handwashing, cleaning and maintenance of facilities). Time constraints and inadequate facilities meant that overall implementation was low. Similarly, Hands Up For Max [9] found that less than two-thirds of schools fully delivered the educational package (lessons, games, posters, homework and other activities) and that the fidelity of the implementation varied across the settings. By contrast, if durability issues can be fixed, PSG are simple to implement and install as they can be quickly attached to facilities by any staff member.

E-bug, Hands Up For Max and Hi Five are multi-component interventions with a heavy lesson plan component that teachers need to deliver [9,11,43]. The PSG in this study are the primary means of communication. This reduces the burden on teachers who are already overstretched [44]. However, for schools to fully embrace a hand hygiene intervention it should be embedded within the curriculum [9,43]. “123” PSG could be extended with additional classroom activities or used to supplement other hand hygiene interventions.

PSG are relatively low cost in economic terms. Unlike Glo-yo [14] which requires the production of new equipment, PSG can be printed on self-adhesive vinyl at low cost. Costs could be further reduced if many schools coordinated printing (for example through academies or health-led initiatives). It is likely though that additional funding will be required as many schools are struggling financially [45].

## 10. Limitations

The limitations of each of the five evaluations are described separately under each evaluation. As the results of each evaluation broadly correspond with the results of the others, it is possible to draw conclusions, albeit tentatively. Some limitations remain and are discussed next.

The idiosyncrasies of the different settings meant that different data collection methods had to be employed for evaluations 2 and 3, making comparisons of settings problematic. 

The PSG were only tested in settings involved in the co-design stage of the study. Though the child participants were sampled across each school and most had not participated in the co-design phase, they still could have felt an affiliation with the PSG that would not be felt in other settings. None of the children from the partner museum participated in the design of “123”. Furthermore, only four settings participated and so it is not clear how generalisable the results are. It is, however, prudent to pre-test interventions before rolling out major campaigns [17]. The evaluation results are sufficiently positive to warrant further evaluations in a large roll-out. 

The evaluations were conducted over a short time period (three months pre and post-installation). From this assessment the immediate impact of the PSG can be identified. Whether hand hygiene is maintained and whether settings continue to implement the designs should ideally be tested two years post-intervention [25]. 

Some flexibility in research design may be necessary when conducting research in schools [26,46]. Ideally, all children in each of the settings would have participated in every evaluation. However, this would have been disruptive to the school day and the children’s learning. The number of participants sampled for each evaluation was balanced against the potential for disruption. Moreover, two schools did not participate in two of the evaluations.

## 11. Future Work

In future work we plan to:

1. improve upon the weaknesses of “123” PSG identified in the evaluation. In particular, the PSG will be modified for comprehension and new solutions for durability issues will be sought;
2. work with new settings (both within the UK and beyond) to consider how “123” can be best adapted for different settings;
3. conduct a larger-scale implementation and evaluation, including a longitudinal evaluation.

## 12. Conclusions

Five evaluations (participant demographic, handwashing frequency, handwashing quality, design persuasiveness and stakeholder views) were conducted to examine the effectiveness and efficiency of the “123” PSG in three UK schools and one museum setting. The results are promising, suggesting that the “123” PSG were substantively effective in increasing hand hygiene in the school settings in which they were designed. This should be validated with further evaluations in more school settings. In the museum setting, the “123” PSG likely increased hand hygiene but the messages need to be simplified for this setting where participants likely only see the designs once. It can also be concluded that PSG are an efficient way of communicating hand hygiene as they require little teacher time to implement and are low cost to produce. PSG can be used either independently of, or in conjunction with, other class-based hand hygiene programs.

This study is important because the toilet space has been largely neglected in prior hand hygiene interventions. This is short-sighted because hand hygiene needs to be communicated at the point-of-decision and not just taught in lessons remote from the activity of handwashing. The findings of this study can be used by researchers and developers of hand hygiene interventions. They could also be used more generally by those developing health-related interventions where messages can be helpfully incorporated into the environment (e.g., doctors’ surgeries and hospitals).

## Figures and Tables

**Table 1:** Participant overview.

	School 1	School 2	School 3	Museum	Total
Design installation	02.04.19	21.03.19	13.03.19	08.03.2019	
Evaluation 1: Participant demographic	n/a—use of publicly available documents				
Evaluation 2: Handwashing frequency	38 days pre and 39 days post, 30.11.2018 to 24.05.2019	n/a—data collection error	20 days pre and 38 days post, 06.03.2019 to 14.06.2019	129 days pre and 39 days post, 21.10.2018 to 19.04.2019	187 days pre and 116 days post
Evaluation 3: Handwashing quality	139 children pre and 138 children post, 14.02.2019 and 12.03.2019	-	-	75 children pre and 69 children post, 04.01.2019 and 09.03.2019	214 children pre and 207 children post
Evaluation 4: Design persuasiveness	43 children, 22.03.2019, 26.03.2019 and 27.03.2019	20 children, 21.05.2019	24 children, 09.05.2019	47 children, 15.04.2019 and 16.04,2019	134 children
Evaluation 5: Stakeholder views	8 staff, 01.05.2019	6 staff, 06.06.2019	8 staff, 09.05.2019	1 staff, 12.06.2019	23 staff

**Table 2:** Setting demographics.

Setting	Location	Age of Children	Size of Setting/No. of Visitors	Ofsted Rating	Catchment
Partner museum	City setting north of England	Target audience is 0–11	302,460 visitors (in 2014)	/	At weekends and during school holidays parents bring children. During weekdays school visits predominate.
School 1	Semi-rural setting East Midlands	4–11	Larger than average^1^ (approx. 420 places)	Good	Majority of children are Caucasian and British. A proportionately below average number^1^ of children are eligible for free school meals. Below average proportion of special educational needs.
School 2	City setting north of England	4–11	Very large (approx. 700 places)	Good	Mostly minority ethnic backgrounds. A proportionately above average number^1^ of children are eligible for free school meals. Above average proportion of special educational needs.
School 3	City setting north of England	4–11	Smaller than average^1^ (approx. 210 places)	Good	High proportion from minority ethnic groups. A proportionately above average number^1^ of children are eligible for free school meals. Slightly below average proportion of special educational needs.

^1^ As reported by Ofsted (precise numbers not available and will fluctuate during the year).

**Table 3:** School hand hygiene product consumption.

Schools	Time Period	Soap Consumption			Dryer Consumption		
		Total	Per Day	% Increase on Baseline	Total	Per Day	% Increase on Baseline
School 1	Pre-installation (20 school days, 06.03.2019 to 29.03.2019	1707 mL	85 mL	-	-	-	-
	Designs installed, 2 Apr						
	Post-installation 1–4 weeks (18 school days, 01.04.2019 to 10.05.2019)	2162 mL	120 mL	41%	-	-	-
	Post-installation 5–8 weeks (20 school days, 13.05.2019 to 14.06.2019)	2600 mL	130 mL	53%	-	-	-
School 3	Pre-installation (38 school days, 30.11.2019 to 06.02.2019)	2750 mL	72.4 mL	-	15 rolls	0.39 rolls	-
	Designs installed, 13 Mar						
	Post-installation I, 1–4 weeks (19 school days, 14.03.2019 to 26.04.2019)	2200 mL	115.7 mL	60%	7 rolls	0.37 rolls	−5%
	Post-installation II, 5–8 weeks (20 school days, 26.04.2019 to 24.05.2019)	1500 mL	75 mL	4%	9 rolls	0.45	15%
	Designs re-installed, 11 Jun						
	Post-installation III, 9–13 weeks (26 school days, 03.06.2019 to 09.07.2019)	2800 mL	107.7 mL	49%	12 rolls	0.46	18%

**Table 4:** Museum soap consumption.

Time Period		Toilets without PSG		Toilets with PSG	
		Girls	Boys	Girls	Boys
Pre-installation (129 days, 21.10.2019 to 28.02.2109)	No. soap changes	9 changes	4 changes	3 changes	2 changes
	Mean no. of days between changes	15 days	38 days	53 days	50 days
Post-installation (39 days, 11.03.2019 to 19.04.2019)	No. soap changes	5 changes	2 changes	3 changes	2 changes
	Mean no. of days between changes	7 days	35 days	19 days	39 days
% increase in soap usage on baseline		53%	8%	64%	22%

**Table 5:** Participant recruitment handwashing quality.

Setting		Girls	Boys	Total
School 1	Pre-installation	69	70	139
	Post-installation	64	69	133
Museum	Pre-installation	41	23	64
	Post-installation	22	34	56

**Table 6:** Participant sample design persuasiveness.

Year Group/(Age)	R/N (4–5)	Year 1 (5–6)	Year 2 (6–7)	Year 3 (7–8)	Year 4 (8–9)	Year 5 (9–10)	Year 6 (10–11)	Year 7 (11–12)	Total
**School 1**									24
Girls	0	0	2	3	2	2	3	0	12
Boys	0	0	5	2	3	1	1	0	12
**School 2**									20
Girls	0	0	10	1	0	0	4	0	15
Boys	0	0	0	0	0	0	5	0	5
**School 3**									43
Girls	0	0	1	7	5	5	10	0	28
Boys	0	0	1	7	2	2	3	0	15
**Museum**									47
Girls	2	2	7	4	5	1	2	2	25
Boys	2	5	4	6	4	0	0	1	22
**Total**	4	7	30	30	21	11	28	3	134

**Table 7:** Interview design and analysis.

Design Persuasiveness	Mapped to (McGuire, 1985)	Mapped to RE-AIM	Interview Question	Analysis
Step 1: Attention	Attention (step 2)	Reach/effectiveness	Q1: When you were in the toilet, did you look at the posters/stickers?	Responses counted.
Step 2: Comprehension	Comprehension of arguments (step 4)	Effectiveness	Q2.1: What do you remember seeing or reading? What did you find out?	Responses verified for comprehension and counted.
			Q2.2: What does 123 mean?	Responses verified for comprehension and counted. Chi-squared statistical tests for age, gender and setting.
Step 3: Acceptance	Acceptance of the arguments (step 7)	Effectiveness	Q3.1: Do you trust the posters/sticker?	Responses counted.
			Q3.2: Why do you/don’t you trust the posters/sticker?	Responses mapped onto a validated scheme of children’s trust criteria [37].
Step 4: Attitude change	Attitude change (step 11)	Effectiveness	Q4.1: Have the posters/stickers changed what you do when you are in the toilets?	Responses counted.
			Q4.2: If so, in what way?	Responses categorised inductively.
			Q4.3: Did you wash your hands just now?	Responses counted and compared with Q4.1.

**Table 8:** Children’s reasons for trusting/not trusting the results mapped onto different constructs of trusts identified by Johnson, Sbaffi and Rowley [37] in the research literature.

Trust Reasons	Definition from [37]	No. of Reasons Given	
		Trust	Not Trust
Usefulness	“The extent to which the user is informed by and can make use of the information”	63	0
Triangulation	“The extent to which the information is consistent with other information on the same topic”	27	0
Authority	“The expertise and standing of the author or organisation responsible for providing the information”	15	0
Credibility	“The believability and impartiality of the information”	6	1
Style	“The way in which the information is presented and written”	2	1
Content	“The core characteristics of the information, such as reliability, accuracy and currency”	0	0
Brand	“Brand indicators and reputation”	0	0
Ease of Use	“The ease of locating, accessing and using the information”	0	0
Recommendation	“Recommendations regarding the information from known person(s)”	0	0
Do not know	The reason why is not known or cannot be explained.	16	3

**Table 9:** Question guide stakeholder interviews.

RE-AIM	Questions	Participants
Closed questions		
Effectiveness	The posters/stickers have encouraged children to wash their hands. Strongly disagree/disagree/neither agree nor disagree/agree/strongly agree/do not know	Head teacher, teachers and museum staff only
Maintenance	The school needs posters/stickers like these in the toilets. Strongly disagree/disagree/neither agree nor disagree/agree/strongly agree/do not know	All staff
Open questions		
Effectiveness	Have the PSG had an impact on the way toilets are used (including unintended consequences)?	All staff
Adoption	What do staff think will be barriers/incentives to adoption?	Head teacher, teachers and museum staff only
Implementation	Have staff/children adapted or wanted to adapt the installation? What do staff think the incentives/barriers to implementation would be in other settings?	All staff
Maintenance	What do staff think are the barriers/incentives to maintaining the PSG long term?	All staff

**Table 10:** Code book.

Theme	Description
Engagement	How children have/have not engaged with the PSG and the impact this has had
Appeal	How the PSG did/did not appeal to children and other audiences
Positioning	The location of the PSG and the connection with other activities in the settings
Durability	Issues affecting the durability of the PSG
Flexibility	The need for PSG to be exchanged and adapted
Economic costs	The financial cost and implications if settings had to self-fund

## Data Availability

This study involves human research participant data and could contain potentially identifying information. The materials used in the present study are available for all academic-based researchers by request. Contact c.m.stones@leeds.ac.uk for more information.
